# Investigation of the separation method of mesophase pitch by two-stage thermal sedimentation

**DOI:** 10.1039/d5ra03324a

**Published:** 2025-08-05

**Authors:** Jingyu Yang, Hui Wang, Jiawei Shang, Yukai Jiao, Ming Li

**Affiliations:** a College of Chemistry and Pharmaceutical Sciences, Qingdao Agricultural University Qingdao 266109 China liming041424@163.com

## Abstract

Mesophase pitch (MP) derived from fluid catalytic cracking (FCC) aromatic-enriched oil exhibits polydisperse molecular architecture, which impairs the spinnability of the resultant carbon fibers. The separation of mesophase pitch has garnered significant attention due to its critical role as a premium precursor for advanced carbon materials. In this study, a mesophase pitch with a softening point of 151 °C was prepared from FCC aromatic-enriched oil through a two-stage thermotreatment process preparation. The anisotropic phase in the mesophase pitch was collected at the bottom of the samples by thermal sedimentation, while the anisotropic phase was collected at the top of the samples. To investigate the temperature-dependent phase separation behavior between the anisotropic phase and isotropic phase, the two phases were systematically characterized using polarized light microscopy, Fourier-transform infrared spectroscopy (FTIR), X-ray diffraction (XRD), thermogravimetric analysis (TGA), and scanning electron microscopy (SEM). Results indicated that the optimal sedimentation effect occurred when the sedimentation temperature exceeded the softening point of mesophase pitch by approximately 50 °C. The superior sedimentation product exhibited enhanced structural ordering with an aromaticity of 0.4354 as measured by FTIR and an order degree of 0.9230, an interlayer spacing of 3.4477 Å as measured by XRD. The ordering degree and thermal stability of the sedimented anisotropic phase were significantly enhanced. During heat treatment, distinct phases of separation occurred between anisotropic and isotropic components, driven by molecular differences: aliphatics migrated upward while condensed aromatic clusters sank. This study showed that thermal sedimentation at the appropriate temperature and time could effectively developed the properties of the mesophase pitch.

## Abstract 2

Mesophase pitch suitable for spinning can be prepare by FCC aromatic-enriched oil under appropriate high-temperature and high-pressure conditions through thermal polycondensation.

## Graphical abstract

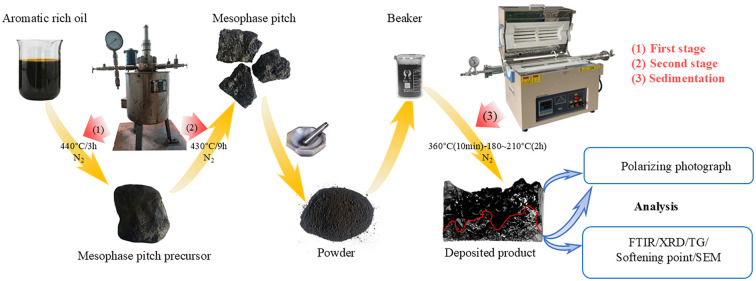

## 1 Introduction

Mesophase pitch, a liquid crystalline material formed through thermal polymerization, consists of ordered stacks of polyaromatic layers.^1–3^ Oil-based mesophase pitch fiber exhibits the advantages of low cost, high carbon yield and excellent axial thermal conductivity. As precursor for advanced carbon materials, MP has been widely applied in aviation, wind turbine blades, automotive lightweight components and high-performance sporting goods in recent years.^4,5^ PAN carbon fibers strength and young's modulus are high, but cost of production is high.^6–8^ The cost of commodity polymers fiber is lower but the tensile strength and modulus are lower than for MP fiber.^9,10^ In addition, carbon fiber reinforced composites have greater advantages in terms of mechanical performance than other reinforced composites.^11,12^ MP can be used in combination with pet blends, low density polyethylene, *etc.*^13,14^ During the preparation process of MP carbon fibers, including the nozzle diameter of the spinning machine and the geometric shape of the 3D printing nozzle, the carbonization process also has a significant impact on the mechanical properties of the carbon fibers.^15–17^ Since the molecular structure of carbon materials is mainly determined by the properties of MP, it's necessary to investigate the thermal polycondensation reaction mechanism for optimizing the preparation of MP with enhanced performance.^18–20^

FCC aromatic-enriched oil is suitable for synthesizing mesophase pitch, because it contains naphthenic, aliphatic groups and polycyclic aromatic hydrocarbons.^21^ The content of polycyclic aromatic hydrocarbons (PAHs) is the critical factor during thermal polycondensation for pitch production.^22–25^ Lou successfully synthesized spinnable mesophase pitch with a yield of 45% and a low softening point of 265 °C through two-stage thermal treatment of FCC-decanted oil.^26^ Through polarized light microscopy and XRD analysis, it was found that the anisotropic domains in oil-based pitch underwent incomplete growth during thermal treatment, resulting in the coexistence of ordered anisotropic structures and disordered isotropic phases.^27^ The roles of light fractions, heavy fractions, and asphaltenes in FCC-derived oil in mesophase pitch formation were systematically analyzed. Gao illustrated that appropriate concentrations of reactive sites on weighty structures promote the polycondensation process.^28^

The advantages of the two-stage thermal treatment process have been recognized in recent years.^29^ MP that prepared by the method contains extensive anisotropic domains and exhibits low softening point, due to reaction rate is slowed down by removing radical lead to balance mesophase content and softening point. Li synthesized mesophase pitch with 100% optical anisotropy and a softening point of 283 °C using a two-stage condensation process catalyzed by boron trifluoride-ethyl valerate complex.^30^ The yield of mesophase was promoted when the light fractions were combined with aryl fraction under high pressure in first stage and the non-mesogens were removed in second stage. The result showed that carbonaceous microcrystalline molecules contained more semi-rigid structures, while parts of alkyl side chains and naphthenic structures were retained during two-stage thermotreatment preparation. Lou investigated the advantage of the mechanism during the two-stage polycondensation.^31^ The appropriate viscosity of bulk mesophase was achieved by removing alkyl side chains and light components promoting development of bulk mesophase significantly. These findings confirmed that the two-stage thermal polycondensation was a simple and effective method for producing mesophase pitch.

Several separation methods are reported that can effectively separate anisotropic structures from mesophase pitch, for example thermal filtration, thermal centrifugation and other physical methods.^32^ Blanco prepared mesophase pitch with anisotropy content of 10–65% from coal-tar pitch. The filter liquor contains 100% isotropy and the filter residue contained 80% anisotropy were obtained *via* thermal filtration separation. It was found that filtrate residue treated by filtration exhibited a better polarizing structure than mesophase prepared by direct thermal polycondensation.^33^ Leuis placed mesophase pitch into a centrifuge, heated it to 300 °C, and centrifuged it at 500 rps. The pitch stratified into two layers: quinoline insoluble content accumulated at the bottom, while suspended particles remained in the top layer. Subsequent analysis confirmed that the bottom phase exhibited anisotropy.^34,35^ Yamada designed a layered heating reactor to produce mesophase pitch. The top layer was a reaction zone that was stably maintained at 430 °C, while the bottom layer as a non-reactive collection chamber. Driven by gravity, the anisotropic phase accumulated at the reactor's base in molten state.^36^ In addition, Kumari measured the viscosity–temperature curve of mesophase pitch and found that low-anisotropy-content pitch showed a broad temperature plateau at 260 °C, whereas the plateau disappeared in high content of anisotropy pitch at the temperature.^37^ Results demonstrated that mesophase pitch could be effectively separated according to the distinct physical properties of anisotropic and isotropic phases at the same temperature.

In this study, mesophase pitch prepared through two-stage thermotreatment process from FCC aromatic-enriched oil was placed into a tube furnace for sedimentation treatment. After the sample melted at 360 °C, the temperature was stabilized in a lower range to ensure no reaction occurred. To investigate the evolution of anisotropic molecular structures, functional groups, and sedimentation mechanisms, FTIR, XRD, TGA, and SEM analyses were conducted on samples which treated at different sedimentation temperatures. The migration mechanism of components in thermal deposition was explained.

## 2 Experimental

### 2.1 Materials

**Fig. 1:**
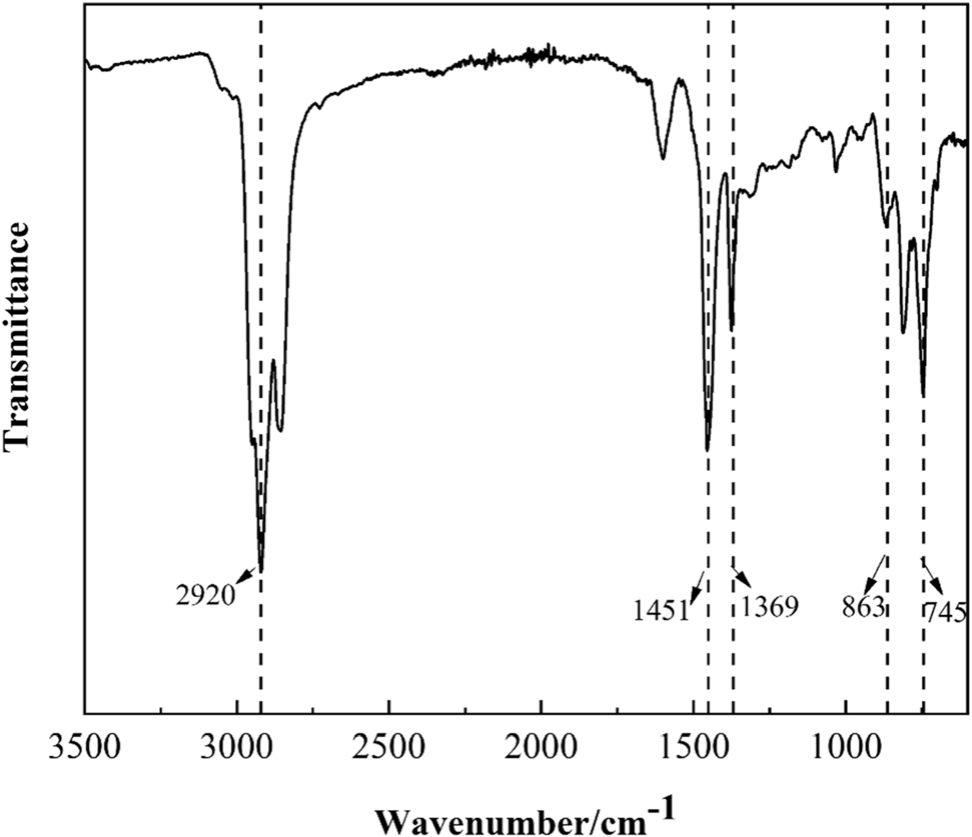
FTIR spectrum of FCC aromatic-enriched oil.

The FTIR spectrum of FCC aromatic-enriched oil used for mesophase pitch preparation was shown in Fig. 1. The strong absorption peaks were observed at 745–863 cm^−1^, representing out-of-plane bending vibrations of aromatic rings. Additionally, peaks at 2920 cm^−1^, 1451 cm^−1^, and 1369 cm^−1^ corresponded to aliphatic C–H bond vibrations. These spectral features demonstrated that the FCC aromatic-enriched oil contained both aliphatic chains and aromatic structures, confirming its suitability as a precursor for synthesizing mesophase pitch.

### 2.2 Preparation of mesophase pitch

In first-stage treatment, FCC aromatic-enriched oil was loaded into a stainless reactor. The reactor was purged with nitrogen three times, then heated to 440 °C for 3 hours under 3 MPa N_2_ pressure to synthesize pitch. In second-stage treatment, the pitch was reloaded into the reactor. After three nitrogen purges (3 MPa), the system was heated to 430 °C for 9 hours under 3 MPa N_2_ to produce mesophase pitch whose softening point was determined to be 151 °C.

### 2.3 Sedimentation treatment

MP obtained using two-stage heat treatment was ground to powder using an agate mortar and sieved through a 300-mesh screen. The MP powder was placed in a glass beaker and loaded into a tube furnace. The tube furnace was purged with N_2_ for 20 min, heated at a rate of 5 °C min^−1^ to 360 °C for 10 min in the nitrogen atmosphere, then cooled down to the sedimentation temperature at a rate of 5°C min^−1^ maintained at 180 °C, 190 °C, 200 °C and 210 °C for 2 h respectively. The sedimentation products were named as MP-180 °C, MP-190 °C, MP-200 °C and MP-210 °C, respectively. The sedimentation samples were vertically divided into two halves, which vertical cross-section diagram was taken as the physical diagram. The top part was denoted by the letter T and the bottom part by the letter B.

### 2.4 Sample characterization

The samples obtained by thermal sedimentation treatment were polished with sandpaper. The polarizing texture of mesophase pitch was observed by polarizing microscope (XP-4030, Limit, China).

FTIR Spectrometer (Nicolet iS10, Thermofisher, USA) was used to characterize the functional group structure under a scanning step size of 4 cm^−1^ and a scanning frequency of 64 frequencies. The aromaticity (*f*_a_) and *ortho*-substitution index (*I*_os_) were calculated as the method in literature.^38^

Mesophase pitch was analyzed by XRD (TD-3700, Dandong Tongda Science & Technology Co., Ltd, China), which a Cu target (*λ* = 0.154056 nm) as radiation source. The layer spacing (*d*_002_), order degree (*O*_g_), and layer stacking height (*L*_c_) were calculated by the method in the literature.^39,40^

The thermal weight loss of mesophase pitch was characterized by a thermogravimetric analyzer (SDT650, Waster, USA) with high-purity nitrogen gas flow of 100 ml min^−1^ and maintained at 1200 °C with a heating rate of 15 °C min^−1^.^41^

The softening points at the bottom of sample was tested by a homemade equipment. The bottom of sedimentation product was ground into powder using an agate mortar and then sieved through a 300-mesh screen. The MP powder was placed in a test tube, and heated under nitrogen protection.

The surface morphology of mesophase pitch was characterized using a scanning electron microscope (JSM-IT500, JEOL, Japan).

## 3 Results and discussion

### 3.1 Analysis of polarizing structure characterization

**Fig. 2:**
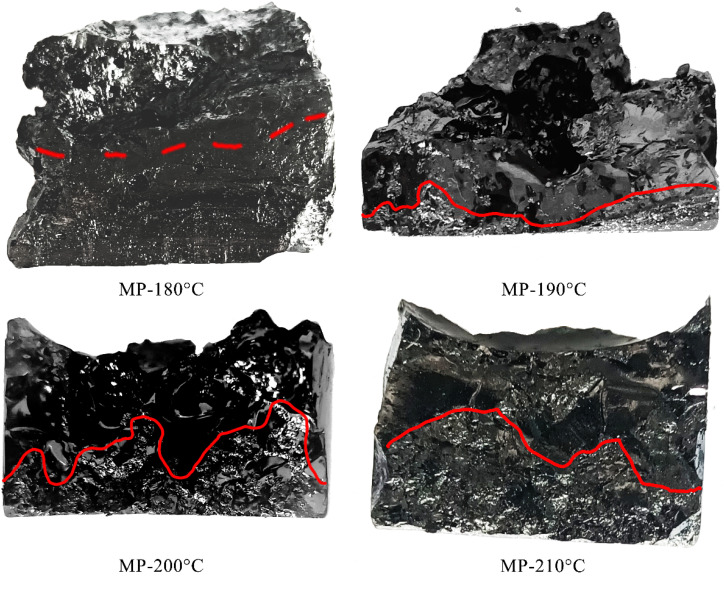
Cross-section photos of sedimentation products.

Fig. 2 displayed the vertical cross-section of samples. The samples were divided into the black bright layer with high isotropic content and the gray layer with high anisotropic content. The boundary was marked with a red line. At 180 °C, the boundary line was irregularly with anisotropic and isotropic components intermixed and no clear separation. This phenomenon was attributed to the high viscosity of the system, which hindered separation during heating.^42^ At 200 °C, distinct gray layer sedimented at the bottom, indicating optimal separation efficiency. This improvement resulted from viscosity of system reduced and density contrast between anisotropic and isotropic phases enhanced. In MP-210 °C, the boundary became irregular and the gray layer thickened, indicating upward diffusion of anisotropic components. These observations demonstrated a strong correlation between separation efficacy and temperature.

**Fig. 3:**
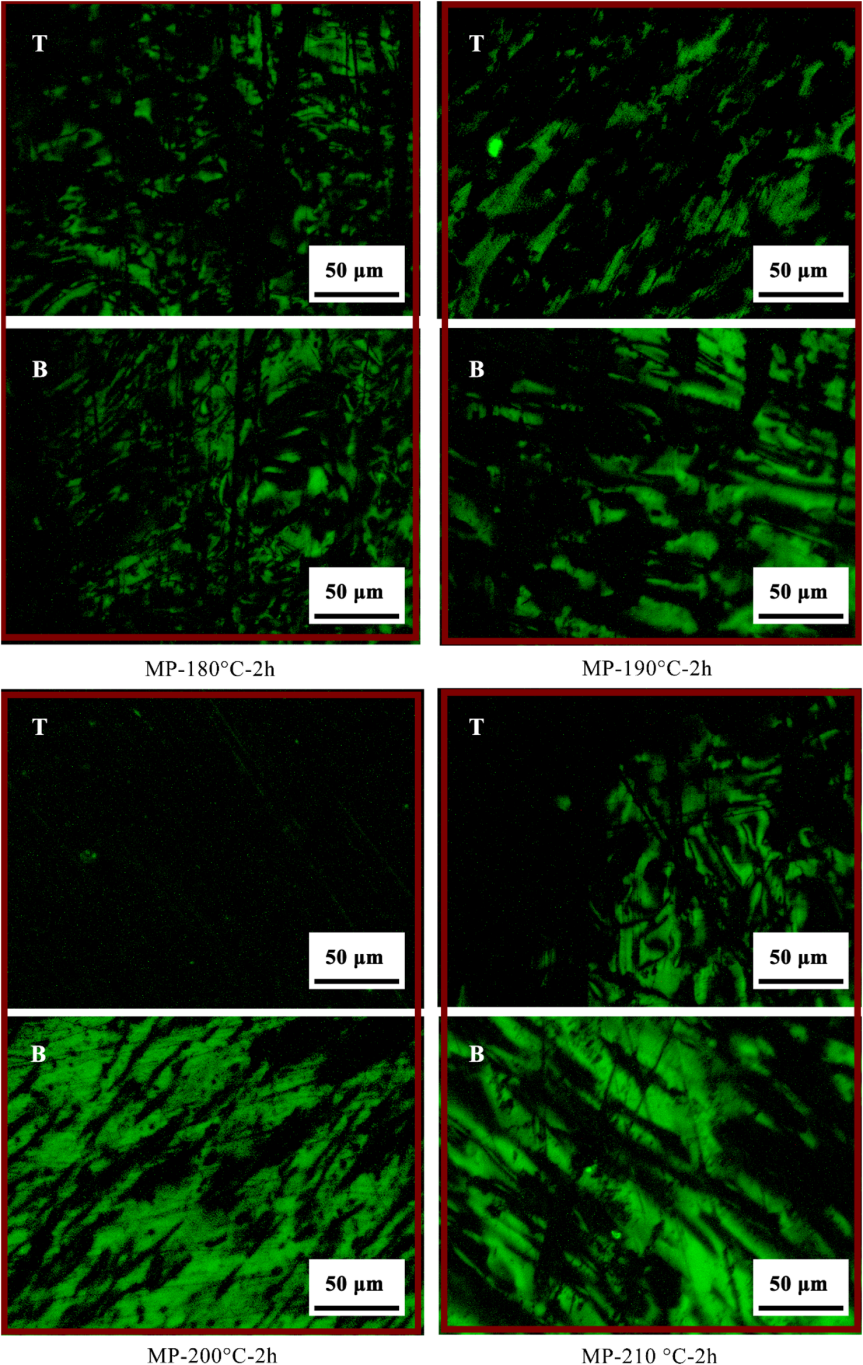
Polarizing pictures of sedimentation products: T, the top of mesophase pitch; B, the bottom of mesophase pitch.

The polarized optical micrographs of sedimentation products were shown in Fig. 3. The polarized structure of the samples was dominated by small domain structure, and the content and morphology of samples were similar at the top and bottom layer, indicating that no significant separation occurred at 180 °C. In 200 °C, the bottom layer exhibited large-basin structures, and the polarizing texture content decreased in the top sample, demonstrating that the sedimentation effect of benzene-ring-based ordered domains was promoted under 200 °C. At 210 °C, polarized structures reappeared in the top region, whereas the content of polarized structures had decreased at the bottom. Anisotropic and isotropic components tended to remix, weakening the thermal sedimentation efficiency. This was attributed to the upward migration of anisotropic phases due to intermolecular forces between aromatic macromolecules and small aliphatic fragments. Notably, at the optimal temperature of 200 °C, microsphere structures at the top sample were significantly reduced, and anisotropic domains at the bottom were improved, confirming temperature-dependent phase separated effectively.

From the above analysis, thermal sedimentation treatment at 190 °C and 210 °C for 2 h resulted in poorly effect. To improve the sedimentation effect at 190 °C and 210 °C, the time of thermal treatment at 190 °C was extended to 3 h, and the duration at 210 °C was shortened to 1 h. The samples were named as MP-190 °C-3 h and MP-210 °C-1 h.

**Fig. 4:**
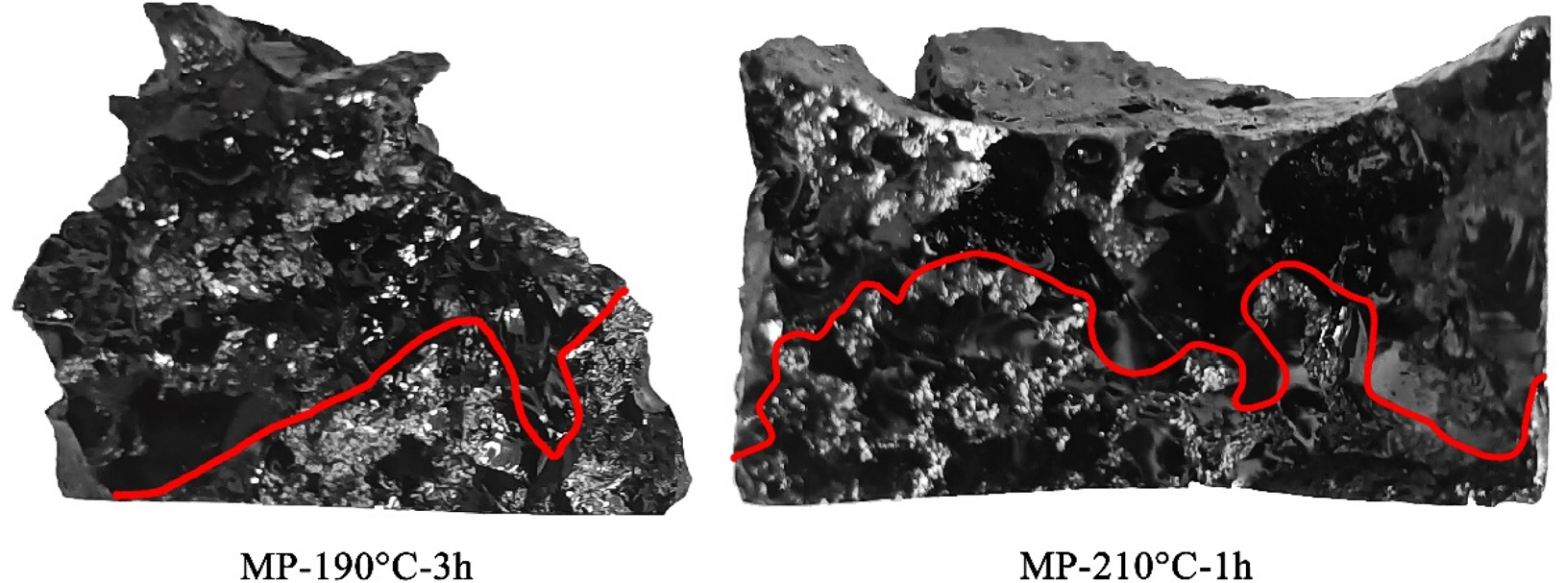
Cross-section photos of sedimentation products.

Fig. 4 showed the sedimentation time shortened to 1 h at 210 °C and the time extended to 3 h at 190 °C. The sedimentation layer of MP-210 °C-1 h became thinner and the boundary line was irregular. It was shown that anisotropy cannot be stably sedimented at the bottom of the sample at 210 °C. Under 190 °C, when the heat treatment time was extended to 3 h, the thickness of the accumulation layer increased slightly. However, the sedimentation effect is still insufficient compared to MP-200 °C. Results showed that at 190 °C and 210 °C heat treatment temperature, the separation effect cannot be obviously improved by extending the heat treatment time. The conclusion was confirmed by shortening the time.

**Fig. 5:**
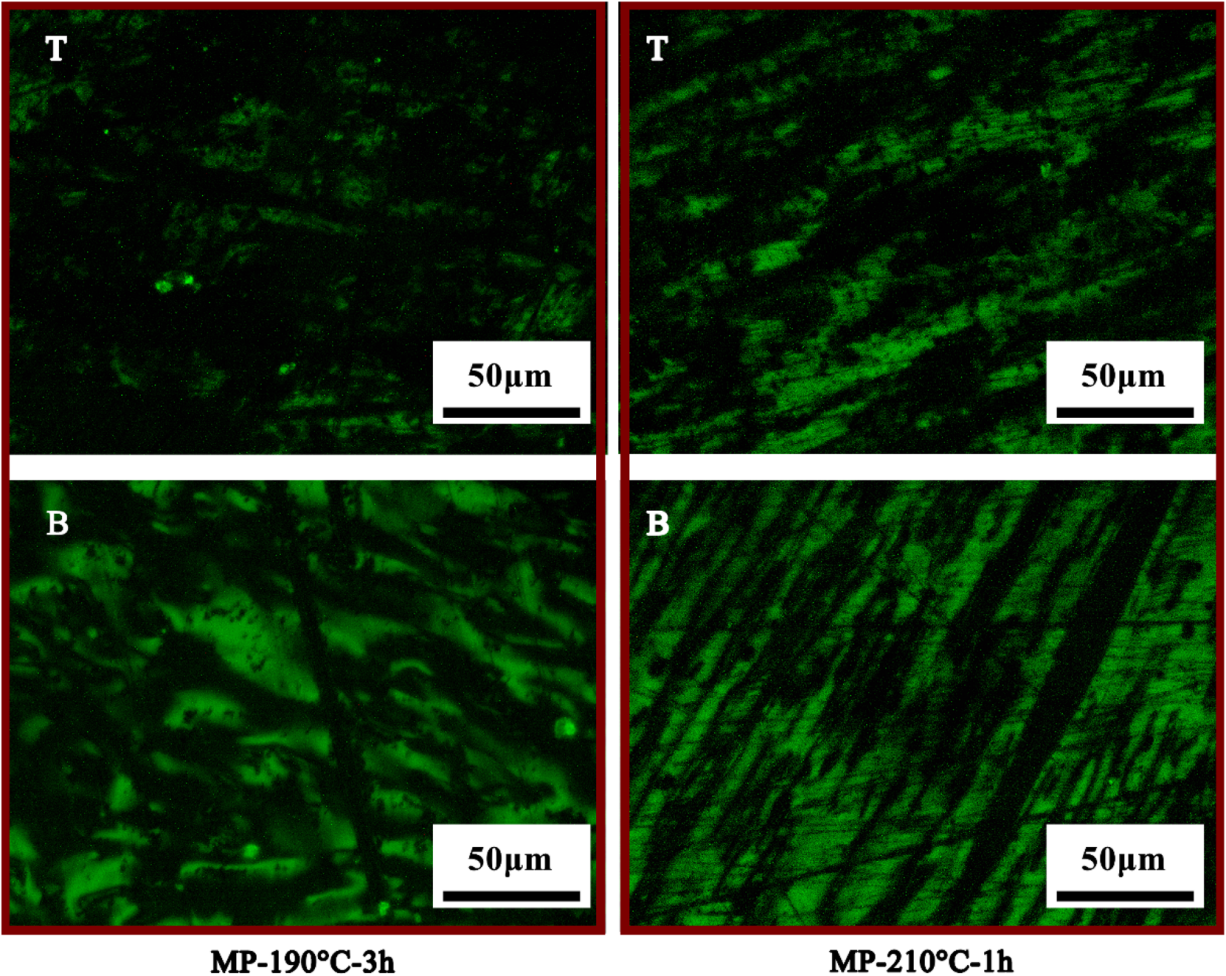
Polarizing pictures of sedimentation products: T, the top of mesophase pitch; B, the bottom of mesophase pitch.

Fig. 5 showed the polarizing structure of MP-190 °C-3 h and MP-210 °C-1 h samples. According to the polarization image of MP-190 °C-3 h, the contain of the anisotropic structure at the top was decreased compared with MP-190 °C. Results showed that the polarized structure morphology of the anisotropic bottom was slightly improved compared to MP-200 °C by extending the time to 3 h at 190 °C. Under 190 °C, it's unsuitable for sedimentation conditions, due to the system viscosity increased. In MP-210 °C-1 h, a large area of anisotropic structures appeared in the top and bottom of samples. Under the treatment temperature of 210 °C, the components were high active during the heat treatment, leading a weak separation effect.

In summary, the effect of thermal sedimentation was influenced by the temperature mainly. After 2 h of treatment temperature at 200 °C, the anisotropy was effectively concentrated at the bottom of sample. Under 190 °C, the sample fluidity was weakened, resulting in a weak sedimentation effect. Above 210 °C, the increase in thermal activity of system is accompanied by evaporating gases form light components, resulting in the mesophase sample cannot achieve effective sedimentation.

### 3.2 Analyze of FTIR characterization

**Fig. 6:**
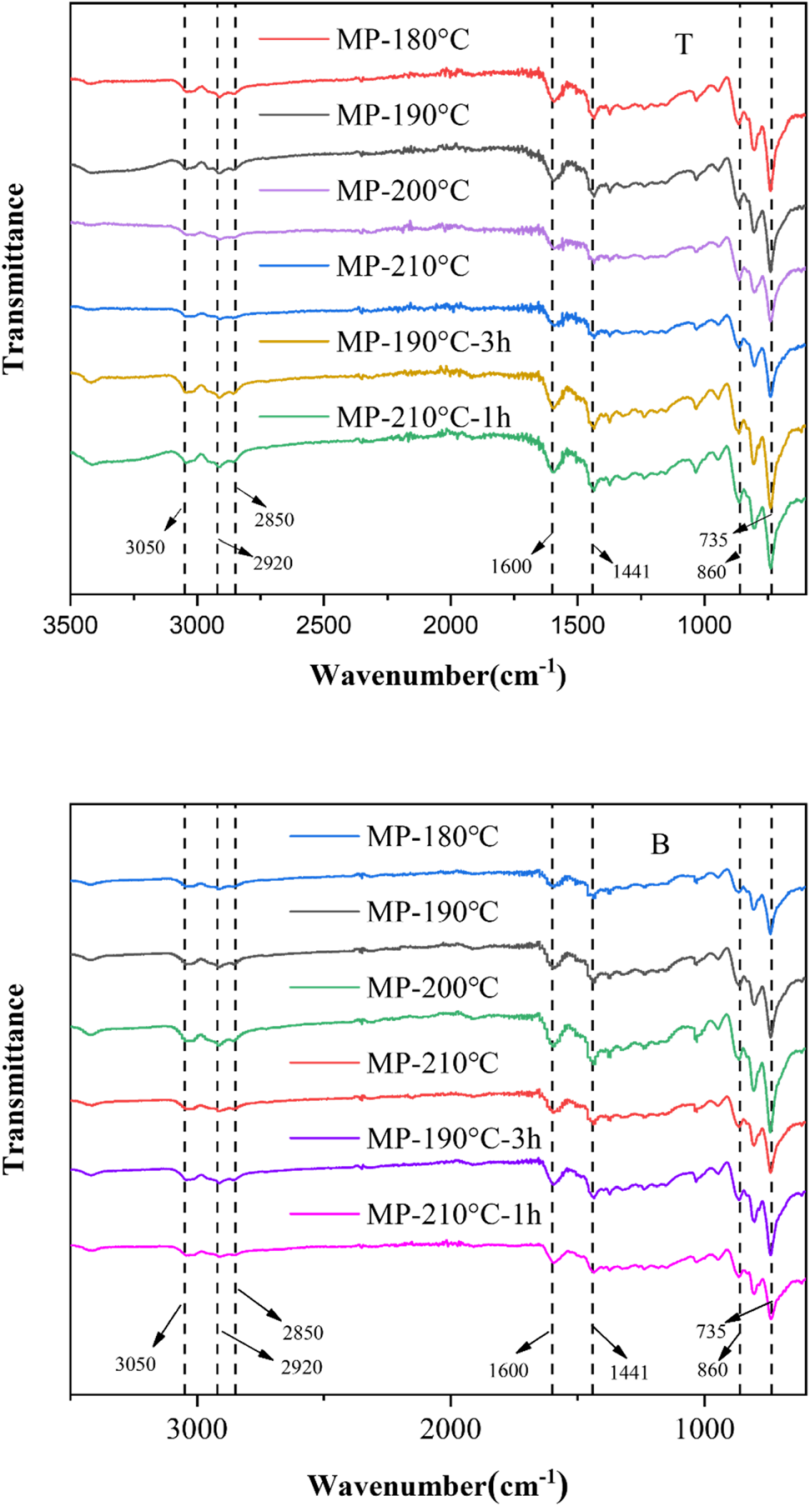
FTIR spectra of sedimentation products: T, the top of mesophase pitch; B, the bottom of mesophase pitch.

Fig. 6 displayed the FTIR spectra of sedimentation products, showing the evolution of functional groups under different sedimentation temperatures. A broad absorption band appeared at 735–860 cm^−1^, corresponding to out-of-plane bending vibrations of aromatic rings. The peak at 3050 cm^−1^ was assigned to aromatic C–H stretching, while the 1600 cm^−1^ peak represented aromatic C=C bond vibrations. The peaks at 2920 cm^−1^ and 2850 cm^−1^ were attributed to aliphatic C–H stretching, and the 1441 cm^−1^ peak indicated aliphatic C–H_2_ deformation.^43^

For the top of MP-180 °C, strong absorption peaks were observed at 735–860 cm^−1^ and 1600 cm^−1^, whereas weak peaks appeared at 2920 cm^−1^, 2850 cm^−1^, and 1440 cm^−1^, suggesting a high aromatic content with minor aliphatic components. For the bottom sample of MP-180 °C, the absorption peak of the aromatic group was weak and the aliphatic absorption peak was higher, indicating that the saturated hydrocarbon tends to fall to the bottom at 180 °C. The absorption peaks at 735–860 cm^−1^ and 1600 cm^−1^ in MP-190 °C-3 h were similar to those at MP-190 °C indicating that the content of aromatic at the bottom of the sample did not obviously change with time prolonging to 3 h. At the bottom of MP-200 °C, the intensity of aromatic peaks at 735–860 cm^−1^ and 1600 cm^−1^ rose significantly, while the aliphatic C–H_2_ peak at 1440 cm^−1^ increased slightly, and the aliphatic C–H peaks at 2920 cm^−1^ and 2850 cm^−1^ remained nearly unchanged. At the top of MP-200 °C, the aromatic peak at 1600 cm^−1^ of the infrared curve decreased. This phenomenon demonstrated that short-chain aliphatics migrated to the top of samples, whereas aromatics and long-chain aliphatics accumulated in the bottom layer during sedimentation. At the bottom of MP-210 °C, the peak intensities of aromatic declined slightly, and aliphatic peaks remained stable, while in the top of MP-210 °C the aromatic content was relatively higher, indicating aromatic groups migrated from bottom to top of the sample. The peak of functional groups which representing aromatic rings in MP-210 °C-1 h were weakened, indicating that the shortening of sedimentation time reduced the content of aromatic groups at the bottom.

The *f*_a_ and *I*_os_ values of sedimentation were summarized in Table 1. The *I*_os_ index at the bottom of MP-180 °C was the highest, representing a high concentration of benzene rings with *ortho*-substituents in the sedimentation product. The *I*_os_ of MP-200 °C was the lowest, suggesting that unsubstituted benzene rings were concentrated at the bottom of sample. Concurrently, the *I*_os_ index at the top of MP-200 °C increased, revealing benzene rings with *ortho*-substituents migrated to top layer. The bottom fraction contained a large concentration of unsubstituted aromatic rings acted as key to form anisotropic phase, while the intermolecular spatial interactions from aliphatic hydrocarbons reduced the order stacking of aromatic domains.

**Table 1:** FTIR parameters of sedimentation products

Samples		*f*_a_	*I*_os_
Top samples	MP-180 °C	0.4269	0.1272
	MP-190 °C	0.4190	0.1901
	MP-200 °C	0.4163	0.2941
	MP-210 °C	0.4177	0.2869
	MP-190 °C-3 h	0.4180	0.2597
	MP-210 °C-1 h	0.4189	0.2354
Bottom samples	MP-180 °C	0.4343	0.3129
	MP-190 °C	0.4350	0.2950
	MP-200 °C	0.4354	0.2796
	MP-210 °C	0.4353	0.3018
	MP-190 °C-3 h	0.4350	0.2837
	MP-210 °C-1 h	0.4348	0.3022

The sedimentation effect of MP-190 °C-3 h was found to have lower *f*_a_ and higher *I*_os_ index compared to MP-200 °C. Results showed that the extension of time at 190 °C temperature conditions had a weak effect on the accumulation of aromatic macromolecular rings, with a small number of saturated hydrocarbons remaining in the bottom layer. This suggested that prolonging the time at 190 °C slightly improved the stacking effect. At the bottom of MP-210 °C-1 h, the *f*_a_ index of the bottom sample decreased to 0.4348 and the *I*_os_ index increased to 0.3022, indicating less accumulation of aromatic macromolecules and more content of saturated hydrocarbons than at the bottom of MP-200 °C. Because the temperature increased to 210 °C, the system of the mesophase pitch was more active. It was found that changing the time effect less on the improvement of thermal sedimentation.

In conclusion, under optimal sedimentation temperatures at 200 °C, unsubstituted aromatic rings accumulated predominantly at the bottom layer, whereas short-chain aliphatic groups and benzene rings with *ortho*-substituents aggregated at the top layer, establishing a thermally driven separation mechanism.

### 3.3 Thermodynamic characterization of sedimentation

**Fig. 7:**
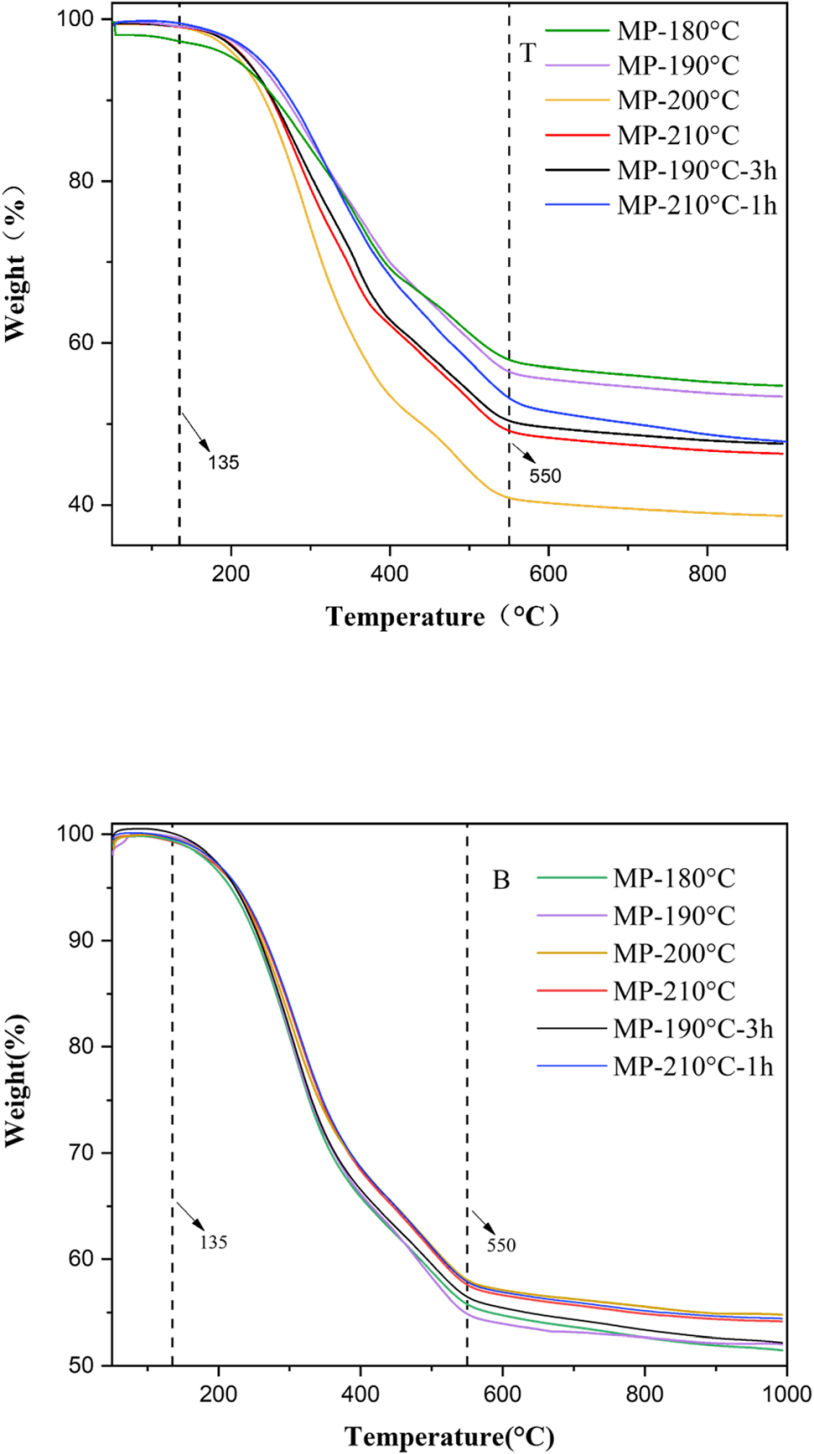
TG curves of sedimentation products: T, the top of mesophase pitch; B, the bottom of mesophase pitch.

The TG curves of thermal sedimentation products at different temperatures were plotted in Fig. 7, demonstrating that the weight loss rate correlated positively with increasing temperature. The pyrolysis process of MP was categorized into three distinct stages. The first stage (below 135 °C) was attributed to water volatilization. During the second stage (135–550 °C), light components were pyrolyzed and removed from the system through cleavage of unstable chemical bonds, with maximum weight loss occurring. The third stage (>550 °C) represented the pyrolysis of recombined components.^44^ Notably, the temperature above 550 °C, the weight stabilized, confirming the dominance of thermally stable macromolecules in the bottom of sedimentation products.

As shown in Fig. 7, no significant mass loss occurred below 135 °C, indicating that evaporation of light fractions was the sole contributor to minor weight changes. The core reactions of mesophase formation that macromolecular cleavage and free radical condensation proceeded predominantly between 135 °C and 550 °C. At the bottom of the sedimentation sample, the weight loss rates of MP-200 °C and MP-210 °C were significantly higher than those of MP-180 °C and MP-190 °C. The MP-200 °C exhibited the highest residual carbon content. The residual carbon content at the top of MP-200 °C was significantly reduced compared to other samples. The weight loss rate of the thermogravimetric curves at the top of samples showed an opposite trend, with the minimum weight loss rate for MP-180 °C and the maximum weight loss rate for MP-200 °C. It was highlighting efficient anisotropic sedimentation under optimal thermal conditions. In contrast, the bottom of MP-180 °C displayed lower final residual carbon content, reflecting polymerization degrees reduced in the bottom of samples. Extending the sedimentation time to 3 h at 190 °C, the residual carbon content of the mesophase pitch was lower than MP-200 °C. It indicated that the 190 °C temperature condition was not suitable for hot settling treatment, because the viscosity of the system was relatively high at lower temperatures, and the extension of the time can only slightly improve the settling effect. There was lower residual carbon content of MP-210 °C-1 h compared to MP-200 °C. It was due to the temperature at 210 °C, the mesophase pitch system was highly active and not suitable for thermal sedimentation, and changing the time did not have a significant effect on the sedimentation effect. It indicated that the two phases were successfully separated through thermal sedimentation, according to differently thermally stability of phases under 200 °C.

**Fig. 8:**
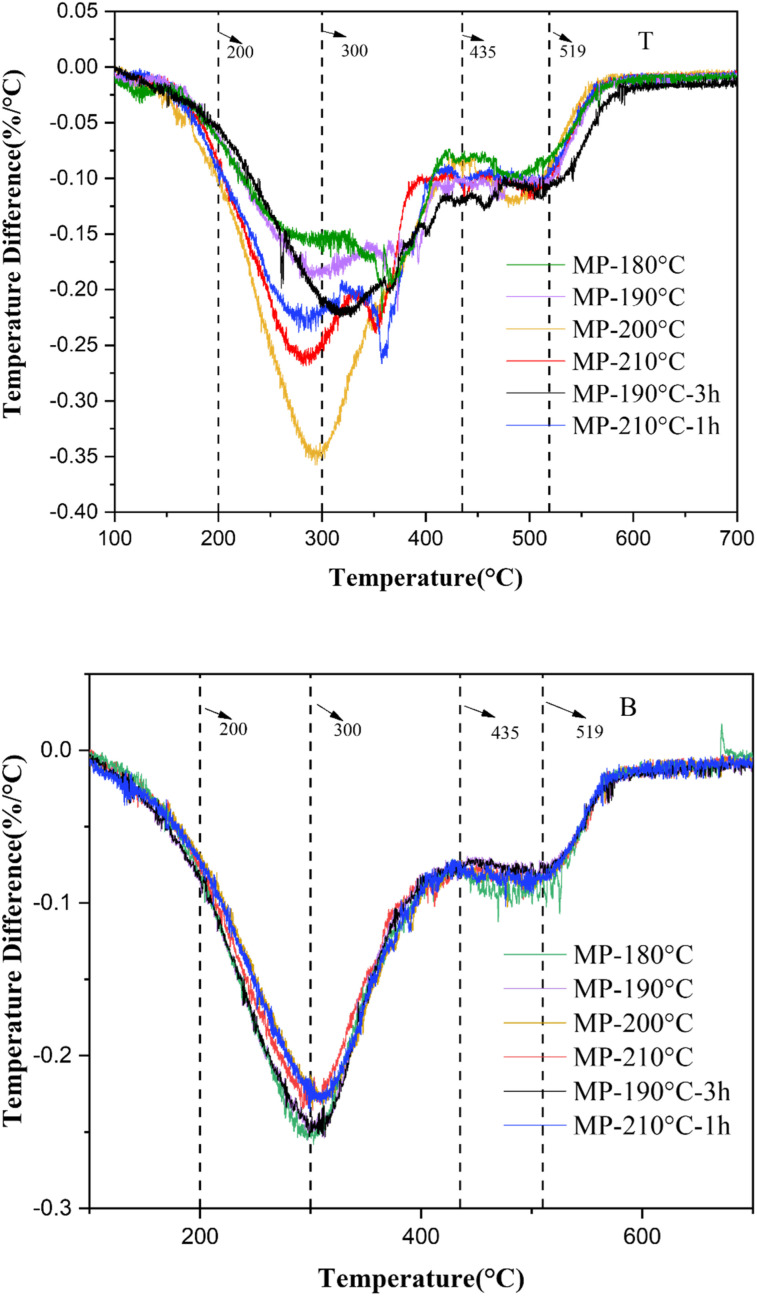
DTG curves of sedimentation products: T, the top of mesophase pitch; B, the bottom of mesophase pitch.

The DTG curves of the samples were shown in Fig. 8 representing the rate of weight loss at different temperatures. Below 300 °C, the rate of weight loss of the sedimentation products increased consistently, and the rate of weight loss below 200 °C was more moderately than the range of 200–300 °C. The bottom products contained saturated hydrocarbon group that tended to crack when the settling temperature was approaching to 200 °C. When the weight loss rate curve exceeded 200 °C, the light component was cracked into gas state and escaped, resulting in the weight loss rate further increased. There was a strong weight loss peak which represented that light component cracking about the temperature of 300 °C. There was an obvious correlation between the sedimentation effect and the pyrolysis of light components when the temperature was 200 °C approximately.

At the bottom of the sedimentation sample, the maximum weight loss peak of MP-180 °C not only had a lower initial weight loss temperature but also had the highest weight loss rate. The initial weight loss temperature was the highest and the weight loss rate was the lowest in MP-200 °C. The DTG curve of the top sample, the weight loss rate of MP-200 °C was the highest maximum, while the weight loss rate of MP-180 °C was the lowest maximum. It indicated that anisotropic phase sedimented with high thermal stability tended to concentrate at the bottom of MP-200 °C. The initial weight loss temperature of MP-210 °C decreased, which was attributed to the light component cracking to produce small molecules. Compared with the DTG curves of MP-200 °C, the samples of MP-190 °C-3 h not only had a large maximum heat loss rate but also had a lower maximum heat loss temperature. This indicated that the effect of prolonging the thermal treatment time at 190 °C on the thermal stability of the bottom sample did not increase significantly with time increased. Compared with the DTG curve of the bottom at MP-200 °C, the maximum rate of weight loss at MP-210 °C-1 h was lower, indicating that the bottom sample was tend to be cracked and less thermally stable in 210 °C-1 h.

In summary, under the thermal sedimentation condition of 200 °C, the thermally more stable components of the mesophase pitch can be sedimented at the bottom of sample, possessing higher residual carbon content, slow cracking rate and low maximum cracking temperature.

### 3.4 Characterization of the softening point of sedimentation

From above conclusions, the migrate tend of component in the sample can be obtained. Through Polarized light microscopy analysis revealing that the polarizing structure at the bottom of MP-200 °C was improved significantly while at the top of MP-200 °C was decreased. Below 190 °C, the high viscosity of the system hindered sedimentation effect of anisotropic structures. Polarization structure tended to move up to the upper layer of the sample when the temperature exceeded 210 °C. Through FTIR analysis revealing that the aromatic gathered at the bottom of the sample, while the aliphatic groups gathered at the top of the sample in MP-200 °C. Under 190 °C, the high viscosity of system hindered the sedimentation of unsubstituted aromatic rings. At 210 °C, the sample were highly thermally active and the aromatics without neighboring substituents migrated from the bottom to the top. Through TG and DTG analysis, the bottom of MP-200 °C were more stable and content fewer chemical bonds can be cracked. The thermal stability of the bottom sample was lower than MP-200 °C when the temperature at 190 °C and 210 °C. It suggested that suitable temperature allows high degree of polycondensation components to be sedimented at the bottom of the sample. In summary, it had proved that anisotropic phase with a high degree of condensation was concentrated at the bottom of the sample, while isotropy containing saturated hydrocarbons and aliphatic-substituted aromatic rings were concentrated at the top of the sample. The characterization of the bottom of samples was analyzed by softening point, XRD spectroscopy, and SEM.

The softening points of the sedimentation products were summarized in Table 2. The softening points of all samples exceeded MP, confirming temperature-dependent sedimentation improved the property of sample at the bottom.

**Table 2:** Softening points of sedimentation products at the bottom

Bottom samples	Softening point (°C)
MP-180 °C	166
MP-190 °C	183
MP-200 °C	227
MP-210 °C	208
MP-190 °C-3 h	190
MP-210 °C-1 h	211

For MP-180 °C and MP-190 °C, the softening points were significantly lower than sedimentation temperatures respectively, because isotropic phases effect the fluidity of mesophase pitch and hindering anisotropic phase accumulation. In contrast, the softening points of MP-200 °C and MP-210 °C surpassed their sedimentation temperatures. Specifically, MP-200 °C exhibited the highest anisotropic content, reflecting optimal polycondensation degree. Above 210 °C, the sedimentation effect deteriorated. Continuous cracking of light fractions generated byproducts, which disrupted the ordered sedimentation of anisotropic phases.

The softening point of MP-190 °C-3 h was increased to 190 °C compared with MP-190 °C, which indicated that the extension of time improved the anisotropic stacking slightly. However, the softening point of MP-190 °C-3 h was lower than that of MP-200 °C. When the treatment time was shortened to 1 h at 210 °C, the softening point was reduced to 185 °C, indicating that the anisotropic stacking degree at the bottom of the sample was lower. The softening point of MP-210 °C-1 h was lower than that of MP-200 °C, indicating that the temperature of 210 °C was unsuitable for thermal sedimentation conditions. It suggested that the settling temperature improved the softening point of the mesophase pitch at 200 °C significantly.

### 3.5 SEM characterization of sedimentation

**Fig. 9:**
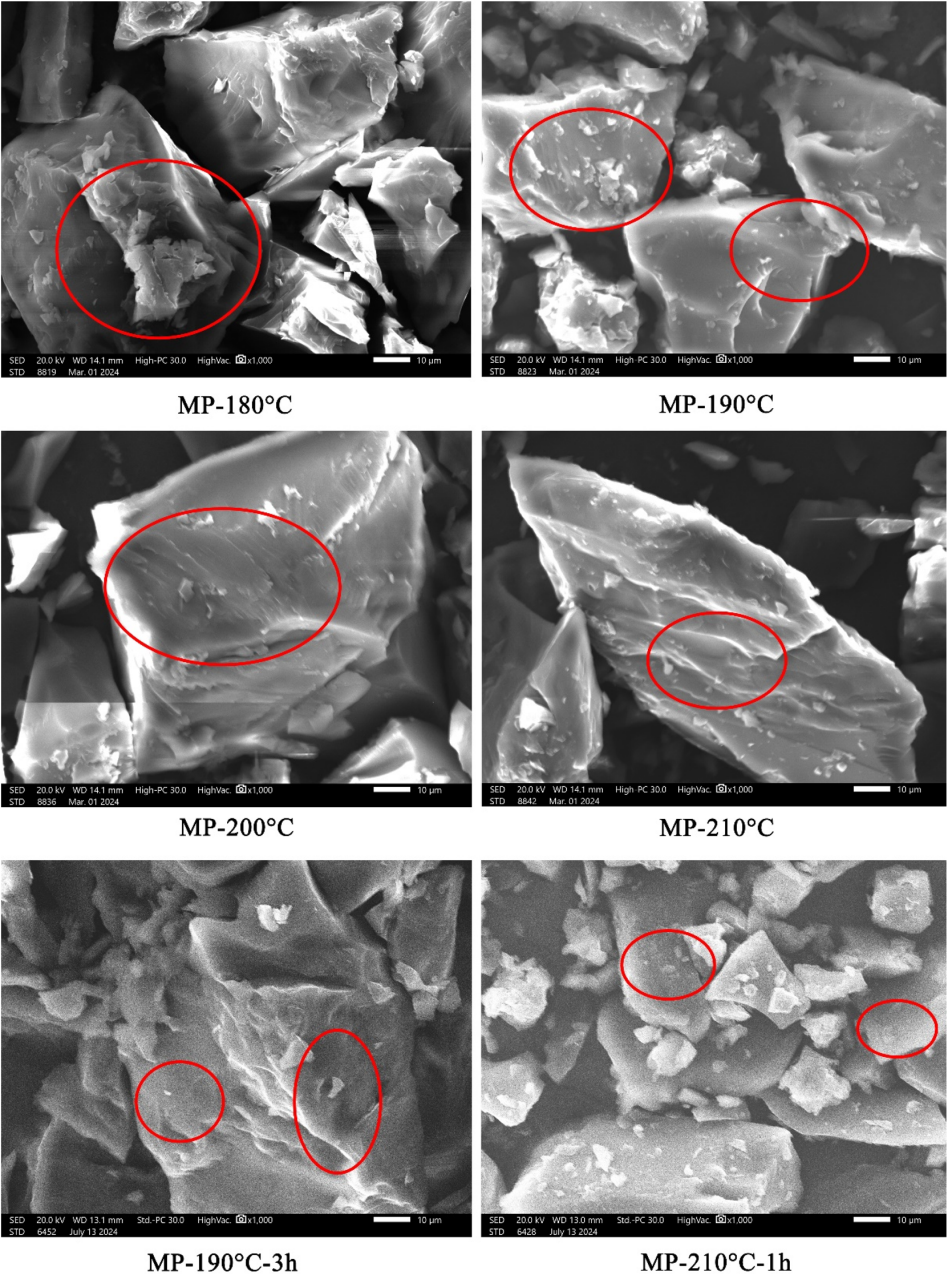
Microscopic pictures of sedimentation products at the bottom.

The microstructural evolution at the bottom of samples was exhibited in Fig. 9. Larger macromolecules sedimented at the substrate's bottom. At 180 °C, the MP system contained both isotropic clusters and partially stacked anisotropic layers. However, the high viscosity of the system hindered sedimentation, resulting in incomplete phase separation. As temperature rising, anisotropic morphology at the bottom gradually homogenized and expanded. MP-200 °C displayed large and coherent anisotropic blocks with smooth layered edges. Isotropic phases were nearly eliminated from the bottom of sample. At 210 °C, isotropic reappeared as a fragmented layer structure. As the processing time of 190 °C was extended to 3 h, the surface of the bottom layer presented a molten state. It explained that the extended time allows the pitch to soften and collected at the bottom under 190 °C. As the processing time of 210 °C was shortened to 1 h, there was a mixture of small molecular lamellae and isotropy in the microstructure picture. It indicated that the shortened time leads to the deterioration of the microstructure state. Scanning electron microscopy proved that 200 °C yielded the highest anisotropic content and perfect structural.

### 3.6 XRD analysis of sedimentation

**Fig. 10:**
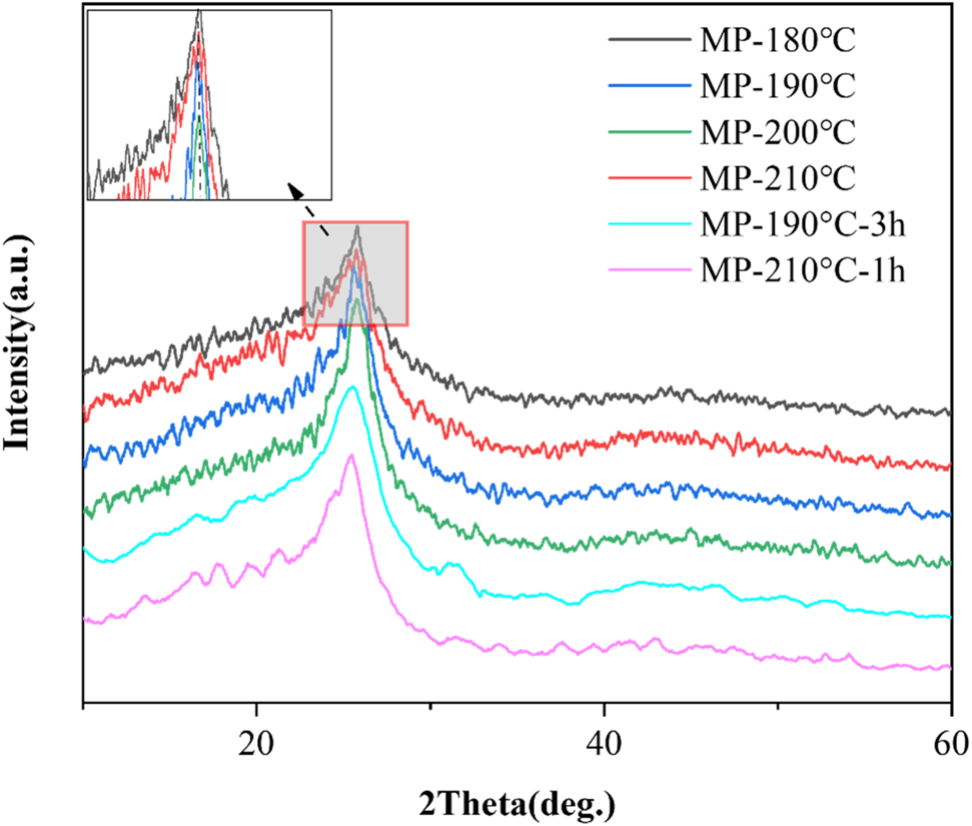
XRD pattern of sedimentation products of mesophase pitch at the bottom.

The experimental curves of the (002) peak in the XRD spectrum of sedimentation products were exhibited in Fig. 10. The MP was a non-graphitic carbon, and its (002) peak showed a wide asymmetric hump. The asymmetry of the (002) peak was influenced by different microcrystalline diffraction peaks overlap and interference of order and amorphous carbon.^45^ The peak of MP-180 °C was slowed down because of less content of ordering carbon rings, indicating that it was difficult for order carbon concentrate at the bottom. The (002) peak of MP-200 °C was the most shape, which indicated that ordered carbon concentrated at the bottom of sedimentation product because the different physical properties of isotropy and anisotropy under 200 °C. While the peak of MP-210 °C was moderative, the accumulation effect of ordered carbon in the sedimentation substrate was worse. In addition to the increase of fluidity, the increase of cracking intensity of light fraction at 210 °C. The peak shape of MP-190 °C-3 h was similar to that of MP-190 °C, indicating that prolonging the sedimentation time at 190 °C had little effect on improving the order degree of sample. Because of the high viscosity of the system at 190 °C, it was not favorable for a high degree of molecule accumulation. The XRD peak patterns of MP-210 °C-1 h were broader and lower than those of MP-200 °C, and the crystal structures of the underlying molecules had a wider range and fewer stacking layers. The results showed that at 210 °C, the effect of shortening the time to improve the crystal structure was limited due to the high temperature that was unfavorable for the stacking of the molecular layer.

The *d*_002_ (interlayer spacing) and *L*_c_ (crystallite height) values were derived from XRD analysis and curve-fitting, as summarized in Table 3. Analysis of the microcrystalline parameters revealed that the diffraction angle in the graphite region initially increased with sedimentation temperature rising and decreased beyond 200 °C. This trend suggested that the sedimentation products contained a high concentration of ordering carbon domains, promoting the planar alignment of polycyclic aromatic hydrocarbon molecules. When the temperature was elevated to 200 °C, *L*_c_ rose from 1.1036 to 1.1630 nm, and the order parameter increased from 0.9189 to 0.9230, confirming enhanced stacking of ordered carbon structures at the bottom layer. During this process, phase separation occurred due to differential fluidity between small-molecular and large-molecular components, leading unsubstituted aromatic rings stacking at the bottom of sample compactly. At 200 °C, *L*_c_ and *O*_g_ of sample declined and *d*_002_ of sample enlarged, indicating that stacking order reduced and interlayer spacing increased. It was because that light component intensified thermal cracking, disrupting the alignment of carbon layers. Compared with MP-190 °C, the ordering degree of MP-190 °C-3 h improved to 0.9227. Compared with MP-200 °C, the ordering degree, and the stacking height were lower. It indicated that the temperature condition of 190 °C were less effective in stacking degree. The stacking height decreased to 1.1333 nm and the layer spacing increased to 3.4768 Å for MP-210 °C-1 h compared to MP-200 °C, indicating that the shortening of the thermal treatment time at 210 °C resulted in a decrease in the stacking of the anisotropic phase.

**Table 3:** Structural parameters of sedimentation products at the bottom

Samples	2*θ*/°	*B*_1/2_/°	*d*_002_/Å	*L*_c_/nm	*O*_g_	*N*
MP-180 °C	25.62	7.30	3.4741	1.1036	0.9189	4.1767
MP-190 °C	25.70	6.98	3.4635	1.1543	0.9224	4.3332
MP-200 °C	25.82	6.93	3.4477	1.1630	0.9230	4.3733
MP-210 °C	25.64	7.20	3.4717	1.1190	0.9200	4.2232
MP-190 °C-3 h	25.72	6.96	3.4608	1.1577	0.9227	4.3452
MP-210 °C-1 h	25.60	7.11	3.4768	1.1333	0.9210	4.2596

These results showed that the optimum sedimentation temperature below 200 °C makes the maximum degree of structure order 0.9230 and the minimum interlayer spacing 3.4477 Å, while deviation from the temperature damages the stacking degree of the anisotropic phase.

## 4 Conclusion

The sedimentation behavior of petroleum-based mesophase pitch was investigated. Mesophase pitches with a softening point of 151 °C and low anisotropic content were successfully synthesized by two-stage thermotreatment preparation process using FCC aromatic-enriched oil as raw material. Results demonstrated that suitable sedimentation temperature improved the properties of sample at the bottom.

Results showed that the sedimentation product was mainly composed of aromatic rings, while the top lay pitch was composed of short-chain aliphatic primarily at the temperature exceeded 50 °C above the softening point. With the fluidity of isotropy increased, sedimentation was occurred effectively under 200 °C due to the different physical properties of isotropic and anisotropic phases. Aromatic rings carrying aliphatic side chains as well as saturated hydrocarbons tended to migrate to the upper layers of MP-200 °C. The anisotropic structure was concentrated at the bottom of the sedimented samples at the appropriate temperature, and the optical structure was improved, with an increase in the softening point to 227 °C, a decrease in the layer spacing to 3.4477 Å, and an increase in the degree of ordering to 0.9230. After extending to 3 h at 190 °C, the sedimentation effect was slightly improved, due to the high viscosity of the system caused by the low temperature, which was unfavorable to the sedimentation of anisotropic phase. The sedimentation effect of MP-210 °C-1 h was similar to that of MP-210 °C. This was due to the high temperature caused by the high thermal activity of the system, which was unfavorable to the sedimentation of anisotropic phase. It indicated that the sedimentation effect was mainly affected by temperature. This work provided a convenient and efficient approach for the separation of anisotropic and isotropic phases in mesophase pitches, which had valuable applications in the high utilization of mesophase pitches and the development of precursors for carbon materials.

## Abbreviations

MP: Mesophase pitch
FCC: Fluid catalytic cracking
FTIR: Fourier-transform infrared spectroscopy
XRD: X-ray diffraction
TGA: Thermogravimetric analysis
SEM: Scanning electron microscopy
PAHs: Polycyclic aromatic hydrocarbons
*f*_a_
: Aromaticity
*I*_os_
: *Ortho*-substitution index
*d*_002_
: Layer spacing
*O*_g_
: Order degree
*L*_c_
: Layer stacking height
*d*_002_
: Interlayer spacing
*θ*
: Diffraction angle
*β*
: Full width at half maxima

## Conflicts of interest

The authors declare that they have no known competing financial interests or personal relationships that could have appeared to influence the work reported in this paper.

## Data Availability

The data supporting this article has been included within the article.
